# The Need of Personalized Medicine in Coping with Stress during Infertility Treatment

**DOI:** 10.3390/jpm11010056

**Published:** 2021-01-18

**Authors:** Małgorzata Nagórska, Bogdan Obrzut, Dariusz Ulman, Dorota Darmochwał-Kolarz

**Affiliations:** 1 Institute of Medical Sciences, Medical College of Rzeszow University, 35-959 Rzeszow, Poland; bogdan.obrzut@gmail.com (B.O.); ddarmochwal@ur.edu.pl (D.D.-K.); 2 Department of Obstetrics and Gynaecology, Pro-Familia Hospital, 35-001 Rzeszow, Poland; ulmandar@gmail.com

**Keywords:** personalized medicine, infertility, psychological factor, stress

## Abstract

The term *personalized medicine* was created for oncological patients, but due to its positive clinical results it is now used in many other fields of medicine, including reproductive medicine. The aim of the study was to determine the level of stress and strategies of coping with stress in patients treated for infertility. The study—using a questionnaire developed by the authors, the Perceived Stress Scale-10 (PSS-10), and the Coping Orientation to Problems Experienced Inventory (Mini-COPE)—was conducted among 456 people from infertile couples. Conclusions: More than half of the studied patients demonstrated a high level of stress. The choice of coping strategies was related to the respondents’ gender and level of stress as well as their experience with assisted reproductive technology.

## 1. Introduction

The term *personalized medicine* (PM) appeared with the development of genomic medicine in 1999 [1]. Personalized medicine, also known as precision medicine, is a novel way for patient’s health management, facilitating optimization of disease treatment. Prophylaxis, diagnosis, and treatment have become more accurate thanks to this concept, while side effects have been minimized, thus saving time and costs on healthcare. By implementing PM, customized treatments can be provided to the benefit of both the patient and the healthcare system [2,3,4].

In the literature on the subject, PM is also referred to as P4 medicine (similar to P5 or P6 medicine), and all of them mean a tailor-made approach in medicine, i.e., treatment individually tailored based on a detailed interview and diagnostics. The term P4 medicine was proposed by biologist Leroy Hood, pointing to four aspects that should be taken into account in the patients’ treatment, i.e., predictive, personalized, preventive, and participatory aspects [1,5,6]. However, the psycho-cognitive sphere was not taken into account in this approach; therefore, the fifth dimension was proposed, and thus P5 medicine was created, which further enriches the patient’s vision with the psychological component that makes each of us different from others [7]. Further, P6 means “public and popular” and highlights the need to share information, e.g., on social media [1].

Initially, personalized medicine (PM) was dedicated to oncological patients and focused on knowledge of genes, proteins, and the environment in diagnostics, prophylaxis, prognosis, treatment, and categorization of patients [8]. This approach gave surprisingly good results in optimizing the therapy in oncology, hence the attempts to use PM in other fields of medicine, including dermatology, endocrinology, cardiology, orthopedics, psychiatry, or in reproductive medicine [2,9,10,11,12,13,14,15]. The implementation of this idea in reproductive medicine offers considerable opportunities, as individual treatment of a specific person or a couple going beyond traditional clinical practice can significantly improve the effectiveness of the treatment [4].

The present study aimed to draw particular attention to the fifth “P”, that is, the psycho-cognitive condition, and specifically to the level of stress and the need to reduce it in the course of infertility treatment. In practice, this area is often overlooked in favor of diagnostics related to the somatic sphere. The WHO recognizes infertility as a social disease with either female or male causes or a mixed cause in both partners. Contemporary medical knowledge confirms that infertility may also have a psychogenic basis, the so-called idiopathic infertility or unexplained infertility [16]. Many authors indicate that infertility treatment is burdened with severe stress; therefore, psychological assistance should be offered according to individual needs, simultaneously with the treatment provided by modern reproductive medicine [17,18].

An individualized approach requires an in-depth recognition of the problem. Hence, standardized questionnaires and tests, a therapeutic interview, or professional psychological assistance can be useful. A considerable challenge in the treatment of infertility is associated with the fact that the participants in the therapy are individual people as a couple. The literature confirms that the attitude to this condition differs in men and women [19,20,21], and the patients’ individual needs resulting from their personality, treatment attitude, relationship, etc. should also be taken into account. In our study, we also took into account the strategies of coping with stress among infertile couples. The aim of the study was to individually determine the level of stress and coping strategies in patients undergoing infertility treatment. It is worth emphasizing that such studies have not been conducted in Poland before.

## 2. Results

### 2.1. Characteristics of the Study Group (n = 456)

In the study participated 456 people from infertile couples: 51.5% (*n* = 235) women and 48.5% (*n* = 221) men. Urban inhabitants constituted 54.4% of the respondents, and 45.6% of the respondents lived in the countryside. The mean age of the respondents was 33.85 years (Standard deviation, SD = 4.76). The youngest person was 24 years old, and the oldest was 52 years of age. The mean age of the women (33.10 ± 4.33 years) was significantly lower (*t* = –3.51; df = 454; *p* = 0.0005) than the mean age of the men (34.64 ± 5.07 years). Four age groups were created based on the values of quartiles. The most numerous was the age group between 30 and 34 years of age constituting 37.3% (*n* = 170). Most of the respondents, 64.7% (*n* = 295), had higher education. The majority of the respondents, 96.3% (*n* = 439), declared the Roman Catholic denomination. The mean duration of the relationship in the study group was 9.14 ± 4.23 years. This period ranged from 2 to 23 years; 19.1% (*n* = 87) of the respondents had been in a relationship up to 5 years. For 39.7% (*n*= 181) of people, the duration of their relationships ranged from 6 to 9 years; 28.1% (*n* = 128) of the respondents were in a relationship for 10 to 14 years; and 13.2% (*n* = 60) of respondents were in a relationship for 15 and more years.

### 2.2. Characteristics/Reproductive History of the Study Participants

In a questionnaire developed by the first author, the respondents were also asked about issues related to the reproductive history of the couple and infertility treatment. The mean time of trying to conceive in the study group was 3.99 ± 2.46 years and ranged from 1 to 15 years. One in three respondents (32.9%, *n* = 150) had been trying to have a baby for 1 to 2 years. The respondents who tried to conceive for 3 to 4 years constituted 34.9%. A smaller percentage of the respondents had been trying to have a baby for 5 to 6 years (19.5%, *n* = 89) or 7 years and more (12.7%, *n* = 58). Primary infertility occurred in 83.1% (*n* = 379) of the respondents. Secondary infertility concerned 16.9% of the respondents.

A total of 232 (50.9%) respondents were aware of their causes of infertility. In the group of those with a diagnosed cause of infertility, 61.6% (*n* = 143) indicated the female factor as the cause of infertility, 25% the male factor (*n* = 58), and 13.4% (*n* = 31) both the female and male factor. 

The most frequently used technique was insemination (29.4%, *n* = 134) and less often the classic in vitro 10.5% (*n =* 48) and in vitro with micromanipulation 8.3% (*n* = 38). Assisted reproductive technology (ART) had not been used in past by 59.9% (*n* = 273) of the respondents.

Most of the respondents (62.3%, *n* = 284) accepted the methods of artificial procreation fully, but one in five persons (20.2%, *n* = 92) did not accept assisted reproductive techniques and was focused exclusively on natural methods. Some methods of artificial procreation were accepted by 17.5% (*n* = 80) of the respondents. The respondents talked about the problem of infertility mainly with their wife or husband (86.0%, *n* = 329). This issue was much less often discussed with friends (30.3%, *n* = 138), family (29.6%, *n* = 135), or medical staff (24.3%, *n* = 111). As many as 5.9% (*n* = 27) of the respondents did not talk to anyone about this topic. Most of the respondents (93.2%, *n* = 425) had not consulted a psychologist. Additionally, a small percentage of respondents reported that they would like such assistance in the future 3.1% (*n* = 14) of people.

The level of perceived stress was assessed with the Perceived Stress Scale-10 (PSS-10). The reliability of the scale was satisfactory (Cronbach’s α = 0.655). The mean score of the intensity of perceived stress was 20.87 ± 5.30 points and ranged from 7 to 38 points. The results of the detailed items are listed in Table 1.

The results of the PSS-10 scale were compared in terms of the sten score, which demonstrated that 4.8% of the respondents experienced a low level of stress (*n* = 22). In the study group, 39.9% of the individuals (*n* = 182) obtained average results in terms of perceived stress. A high level of stress was found in 55.3% of the respondents (*n* = 252). Women experienced a higher level of stress more often (63.0%) than men (47.1%), and the difference was statistically significant (*p* = 0.0006). 

Strategies for coping with stress were defined with the Mini-COPE scale, and the detailed results are presented in Table 2.

The preferred coping strategies in difficult situations were active coping (2.26 points), planning (2.13 points), and acceptance (1.86 points). The least often used strategies in difficult situations were behavioral disengagement (0.77) and substance use (0.44 points); see Table 3.

The level of stress was found to influence coping strategies in difficult situations. People experiencing higher levels of stress were more likely to choose the following strategies: self-blame (rho = 0.273), behavioral disengagement (rho = 0.132), venting (rho = 0.107), and denial (rho = 0.157). People experiencing lower levels of stress more often chose a strategy related to positive reframing (rho = −0.147) and sense of humor (rho = −0.141). The choice of coping strategies in difficult situations in the group of women and generally in the group of respondents was related to the level of stress in a similar way. A higher level of stress generated a choice of coping strategies related to denial (rho = 0.205), behavioral disengagement (rho = 0.182), and self-blame (rho = 0.373). The lower level of stress in women determined the choice of coping strategies based on positive reframing (rho = −0.220), sense of humor (rho = −0.187), and use of emotional support (rho = −0.130). In the group of men, a higher level of stress influenced coping through venting (rho = 0.177) and self-blame (rho = 0.153), see Table 4. 

In the next step, the differences between the level of perceived stress were verified (low/average vs. high, i.e., two groups because few people had “low” score). Generally, people with a high level of stress more often chose the self-blame (1.40 points) strategy than people with low/average levels of stress (1.05 points). Additionally, the respondents with a high level of stress more often decided to choose the strategy of self-distraction (1.88 points; *p* = 0.0275). People with low/average levels of stress coped with difficult situations more often through positive reframing (1.87 points) and sense of humor (0.99 points). The analysis of the study material showed that the influence of stress on coping strategies in difficult situations was visible mainly among women. The women with high levels of stress more often chose denial (1.11 points), behavioral disengagement (0.86 points), and self-blame (1.47 points). In the group of women with low/average stress levels, the following coping strategies were preferred: positive reframing (1.91 points), sense of humor (0.94 points), use of emotional support (1.82 points), and use of instrumental support (1.92 points). 

In the group of men, the level of stress was found to be influenced only by the self-blame strategy—it was more often chosen by men with a high stress level (1.30 points) than men with low/average levels of stress (1.08 points); see Table 5. 

In the further part of the analysis, the patients’ experience of using ART procedures was also considered, and a comparison was made between the group of patients who experienced ART and those without such an experience. In the group of people using the ART procedures, higher levels of stress translated into more frequent use of the sense of humor strategy (*p* = 0.0057) and more frequent self-blame (*p* = 0.0023). People who had a higher stress level used psychoactive substances less often (*p* = 0.0065). In the group of people who did not use any ART procedures, positive reframing was more often chosen by people with low/average stress (*p* = 0.0266). People with high stress more often used denial (*p* = 0.0078), behavioral disengagement (*p* = 0.0063), and self-blame (*p* = 0.0001) (Table 6).

## 3. Discussion

Diagnosing the cause of infertility in a couple takes time, especially when the causes are complex and involve both partners. In some cases, the diagnosis and long-term treatment do not bring the expected result, which significantly affects the mental health of the partners. Some couples will have their own offspring, but a significant number will remain childless unintentionally.

The present study attempted to determine the level of stress as well as coping strategies in patients in infertile relationships. The conducted analysis confirmed the high level of stress in over half of the respondents. Those respondents more often selected the strategies of self-blame and self-distraction as a method of reducing stress. On the other hand, people with low or average levels of stress chose the strategies of positive reframing and sense of humor. Moreover, it has been demonstrated that the selection of a stress reduction strategy depends on the level of stress and the gender of the respondents and that higher levels of stress are more common in women than in men, which was also confirmed in the studies by other authors [19,20,21,22].

Stress reduction strategies differed by gender. Women with high levels of stress applied the strategies of denial and then the strategies of behavioral disengagement and self-blame more often, which was in line with the study by Wu et al. [23]. In the group of women with low or average stress levels, the following coping strategies were preferred: positive reframing, sense of humor, and use of emotional or instrumental support. In the case of men with a high stress levels, the most frequent choice was the self-blame strategy.

Many authors, using various research tools, defined the strategies of coping with stress in infertile couples. Mohammadi showed that women most often chose an emotion-focused coping style, while men chose a problem-focused coping style [24]. A study of Karaca indicated that the emotion-focused coping methods (talking with husbands or friends), religious rituals, and an avoidance (social withdrawal) coping strategy were preferred by infertile women. They also often used alternative medicine methods to support the treatment of infertility [25]. Addtionally, in the similar study of Alosami, women were prone to be more religious and talk to others about this problem. Moreover, they were more likely than men to use alternative medicine methods. Women also had higher levels of stress and greater desire to have children than men [26]. On the other hand, the study of Pottinger showed that women with higher levels of stress chose social distance (avoidance) and self-blame. Women were more prone to social depreciation and suffered more from the lack of children than men. To deal with the problem, both women and men used conventional medicine methods and tried to think positively [27]. On the other hand, Babore showed that infertile men with higher levels of stress most often adopted the strategies of self-blame and avoidance [28].

Many of the above-cited authors indicated infertility as the cause of a significant mental burden and agreed that systemic solutions and procedures should be introduced that would allow for the simultaneous recognition of the stress level and implementation of appropriate solutions adequately to the individual situation [18,24,27,28,29,30]. Patel pointed to the important role of mental health experts in identifying and reducing stress and the need of integrating psychological care into a fertility treatment protocol. He also highlighted the importance of the use of psychotherapy in treating infertility in patients at risk [31]. Rooney claimed that psychological support for infertile women reduces anxiety and depression and may contribute to a higher treatment success rate [18].

It has been proven many times that infertility causes many psychological problems [32], increases the level of stress [18,29,33], and can also lead to depression [23,32]. Despite this, even the largest scientific societies of reproductive medicine such as the European Society for Human Reproduction and Embryology (ESHRE) and the American Society for Reproductive Medicine (ASRM) do not propose obligatory psychological counselling procedures for patients with infertility [18]; so, the question of mental health in infertile couples is often overlooked in the treatment process. Our study also showed that the majority of patients did not use psychological help; similar results were obtained by Pash, who also emphasized that more attention should be paid to the psychological sphere of infertile patients [29]. Many authors emphasize that suffering from gynecological disease frequently results in the elevated stress level [34,35,36]. Such disease occurred also in some of the women surveyed in our study, and it was indicated by them as the cause of infertility.

## 4. Materials and Methods

The cross-sectional study was conducted in southeastern Poland in gynecological outpatient clinics among randomly selected individuals from infertile relationships. Data were collected from June 2019 to February 2020. People meeting the following inclusion criteria were invited to participate in the survey: diagnosed infertility as defined by the World Health Organization (WHO) (i.e., as a failure to achieve a clinical pregnancy after 12 months or more of regular unprotected sexual intercourse [37]), over 18 years of age, and informed consent to participate in the study. The exclusion criterion was failure to meet the above requirements. The Polish Gynecological Society (PTG) states that about 1.5 million couples have fertility issues in Poland [37]. The sample size was computed using the G*Power 3.1.9.2 program (Heinrich Hein University, Düsseldorf, Germany). The minimum required sample size of a group was 383, and 456 people took part in our study. All participants were notified about the possibility of withdrawing from the study at every stage.

### 4.1. Ethical Consideration

The study was conducted in accordance with the Declaration of Helsinki for medical research. The authors obtained permission of the Bioethical Committee at the University of Rzeszow (resolution number 2018/04/03). 

### 4.2. The Course of the Study

Prior to the implementation of the study, the medical facilities approved its design. The couples were informed about the possibility of participating in the study and its purpose during the registration process just before an appointment with a gynecologist. Each participant expressed informed consent to participate in an anonymous study. Five hundred questionnaires were distributed and 456 fully completed questionnaires were returned.

The paper-and-pencil study was conducted in compliance with the conditions of voluntary participation and anonymity. Three measurement tools were used in the analysis: a proprietary survey for collecting socio-demographic data and reproductive history, the Perceived Stress Scale (PSS-10), and an inventory to measure coping strategies with stress (Mini-COPE).

The questionnaire developed by the first author consisted of 11 questions: The items in the questionnaire referred to sex, age, education, place of residence, attitude to religion, duration of the current relationship, duration of trying to conceive, type of infertility, applied ART methods, acceptance of the situation, and the need for psychological assistance. The level of stress related to the life situation was assessed using the PSS-10 questionnaire and the coping strategies with the Mini-COPE inventory tool. The study used the Polish versions of the PSS-10 and Mini-COPE, adapted and validated by Zygfryd Juczyński and Nina Ogińska-Bulik [38]. 

### 4.3. 10-Item Perceived Stress Scale (PSS-10)

The PSS-10, Perceived Stress Scale developed by Cohen et al., was used to assess the intensity of stress related to one’s life situation over the last month [39]. The PSS-10 scale includes 10 items and allows the determination of subjective feelings related to personal problems and events and the ways of dealing with them. Answers are given on a scale of 0–4 (0—never, 1—almost never, 2—sometimes, 3—quite often, 4—very often). The PSS-10 consists of 6 positively (items 1, 2, 3, 6, 9, and 10: positive factor) and 4 negatively (items 4, 5, 7, and 8: negative factor) worded items. Negatively worded items were re-coded during analysis. The overall score is the sum of all points and ranges from 0 to 40. The higher the final score, the greater the perceived stress. The general index after conversion to standardized units is interpreted according to the properties characterizing the sten scale. A final score ranging from 1–4 sten is defined as low, within 5–6 sten is defined as average, and 7–10 sten is defined as high.

### 4.4. Mini-COPE Inventory

Strategies of coping with stress were examined with the Mini-COPE questionnaire developed by Carver et al. [40]. The questionnaire consists of 28 statements that are a part of 14 strategies for coping with stress. AC: active coping (items 2, 7), P: planning (items 14, 25), PR: positive reframing (items 12, 17), A: acceptance (items 20.24), SH: sense of humor (items 18, 28), R: religion (items 22, 27), UES: use of emotional support (items 5, 15), UIS: use of instrumental support (items 10, 23), SD: self-distraction (items 1, 19), D: denial (items 3, 8), V: venting (items 9, 21), SU: substance use (items 4, 11), BD: behavioral disengagement (items 6, 16), and SB: self-blame ( items 13, 26). The inventory is based on a 4-point Likert scale (0 to 3), where 0 = I usually don’t do this at all, 1 = I usually do this a little bit, 2 = I usually do this a medium amount, and 3 = I usually do this a lot. Each of the 14 strategies is scored separately by adding the points for the two statements and dividing the sum by 2. The higher the score on the scale, the greater the tendency to use the strategy [39].

Statistical analysis was performed using the program IBM SPSS Statistics 20. The results obtained were subjected to statistical analysis using descriptive statistics methods (M: arithmetic mean, Me: median, SD: standard deviation), the Pearson chi-squared independence test, and the Mann–Whitney test. The level of significance was adopted at *p* < 0.05.

## 5. Conclusions and Clinical Implications

More than half of the patients in infertile relationships demonstrated a high level of stress, with women having a significantly higher stress load. The choice of coping strategies depended on the respondents’ gender and ART experience as well as the degree of their stress. In the group of women with high levels of stress, strategies such as denial, behavioral disengagement, and self-blame prevailed, while in men only self-blame. Patients with higher levels of stress who underwent ART procedures most often used the sense of humor strategy, and those who did not have such experience more often used the denial, behavioral disengagement, and self-blame strategies.

Strategies of coping with stress seemed to be widely varied, i.e., there was no single pattern for all patients. This clearly indicates the urgent need of an individual approach in diagnosis and treatment of psychological problems. Psychological support should be offered to the patients depending on their needs and preferences.

The very low percentage of respondents who had consulted a psychologist indicates that patients should be more strongly informed about the possibility and/or need of such support, depending on their needs and preferences. In practice, standardized tools should be used to assess the mental state of infertile patients at the beginning of treatment and, in justified cases, individual and/or group psychotherapy should be offered. Identifying individual ways of coping with stress would potentially help to find an optimal therapeutic strategy.

### Limitations

The study was conducted only in a group of infertile couples, which does not allow comparison with the level of stress with a group of fertile people. Moreover, a certain limitation may result from the fact that the study was conducted only in private healthcare centers.

## Figures and Tables

**Table 1:** The level of perceived stress Perceived Stress Scale-10 (PSS-10)—the results of individual items (0–4 points scale) and the overall result (0–40 points scale).

Items	M	Me	SD	Min.	Max.
1. In the last month, how often have you been upset because of something that happened unexpectedly?	2.50	2	0.94	0	4
2. In the last month, how often have you felt that you were unable to control the important things in your life?	1.86	2	1.07	0	4
3. In the last month, how often have you felt nervous and “stressed”?	2.70	3	0.97	0	4
4. In the last month, how often have you felt confident about your ability to handle your personal problems?	2.20	2	1.39	1	7
5. In the last month, how often have you felt that things were going your way?	1.97	2	1.20	1	7
6. In the last month, how often have you found that you could not cope with all the things that you had to do?	1.77	2	1.03	0	4
7. In the last month, how often have you been able to control irritations in your life?	1.93	2	1.05	1	4
8. In the last month, how often have you felt that you were on top of things?	1.90	2	0.92	1	4
9. In the last month, how often have you been angered because of things that were outside of your control?	2.14	2	0.99	0	4
10. In the last month, how often have you felt difficulties were piling up so high that you could not overcome them?	1.89	2	1.09	0	4
PSS-10 (0–40 points scale)	20.87	20	5.30	7	38

M: arithmetic mean, Me: median, SD: standard deviation.

**Table 2:** Strategies for coping with difficult situations (Mini-COPE)—the results of individual items.

Scale 0–3 Points	M	Me	SD	Min.	Max.
1. I’ve been turning to work or other activities to take my mind off things.	1.98	2	0.84	0	3
2. I’ve been concentrating my efforts on doing something about the situation I’m in.	2.21	2	0.65	0	3
3. I’ve been saying to myself "this isn’t real".	0.86	1	0.83	0	3
4. I’ve been using alcohol or other drugs to make myself feel better	0.46	0	0.69	0	3
5. I’ve been getting emotional support from others.	1.70	2	0.86	0	3
6. I’ve been giving up trying to deal with it.	0.80	1	0.73	0	3
7. I’ve been taking action to try to make the situation better.	2.31	2	0.66	0	3
8. I’ve been refusing to believe that it has happened.	1.09	1	0.88	0	3
9. I’ve been saying things to let my unpleasant feelings escape.	1.45	1.5	0.86	0	3
10. I’ve been getting help and advice from other people.	1.64	2	0.87	0	3
11. I’ve been using alcohol or other drugs to help me get through it.	0.42	0	0.68	0	3
12. I’ve been trying to see it in a different light, to make it seem more positive.	1.76	2	0.79	0	3
13. I’ve been criticizing myself.	1.30	1	0.88	0	3
14. I’ve been trying to come up with a strategy about what to do.	2.10	2	0.70	0	3
15. I’ve been getting comfort and understanding from someone.	1.78	2	0.81	0	3
16. I’ve been giving up the attempt to cope.	0.74	1	0.75	0	3
17. I’ve been looking for something good in what is happening.	1.83	2	0.72	0	3
18. I’ve been making jokes about it.	1.40	1	0.90	0	3
19. I’ve been doing something to think about it less, such as going to movies, watching TV, reading, daydreaming, sleeping, or shopping.	1.67	2	0.86	0	3
20. I’ve been accepting the reality of the fact that it has happened.	1.86	2	0.74	0	3
21. I’ve been expressing my negative feelings.	1.41	1	0.81	0	3
22. I’ve been trying to find comfort in my religion or spiritual beliefs.	1.33	1	0.96	0	3
23. I’ve been trying to get advice or help from other people about what.	1.56	2	0.87	0	3
24. I’ve been learning to live with it.	1.87	2	0.74	0	3
25. I’ve been thinking hard about what steps to take.	2.16	2	0.69	0	3
26. I’ve been blaming myself for things that happened	1.19	1	0.86	0	3
27. I’ve been praying or meditating.	1.38	1	1.00	0	3
28. I’ve been making fun of the situation.	0.41	0	0.62	0	3

M: arithmetic mean, Me: median, SD: standard deviation, COPE: Coping Orientation to Problems Experienced Inventory.

**Table 3:** Strategies for coping with difficult situations (Mini-COPE).

Scale 0–3 pts.	Mean	Me	SD	Min.	Max.
AC	2.26	2	0.56	0	3
P	2.13	2	0.59	0	3
PR	1.80	2	0.65	0	3
A	1.86	2	0.63	0	3
SH	0.91	1	0.61	0	2.5
R	1.36	1.5	0.92	0	3
UES	1.74	2	0.76	0	3
UIS	1.60	1.5	0.77	0	3
SD	1.82	2	0.69	0	3
D	0.97	1	0.73	0	3
V	1.43	1.5	0.62	0	3
SU	0.44	0	0.65	0	3
BD	0.77	1	0.61	0	3
SB	1.24	1	0.76	0	3

AC: active coping, P: planning, PR: positive reframing, A: acceptance, SH: sense of humor, R: religion, UES: use of emotional support, UIS: use of instrumental support, SD: self-distraction, D: denial, V: venting, SU: substance use, BD: behavioral disengagement, SB: self-blame.

**Table 4:** Strategies for coping with difficult situations (14 scales) and the perceived level of stress.

Mini-COPE	General (*n* = 456)		Women (*n* = 235)		Men (*n* = 221)	
	rho	*p*	rho	*p*	rho	*p*
AC	0.001	0.9895	−0.012	0.8505	−0.001	0.9854
P	−0.004	0.9297	−0.001	0.9906	−0.031	0.6505
PRE	−0.147	0.0017	−0.220	0.0007	−0.066	0.3322
A	−0.053	0.2630	−0.128	0.0505	0.017	0.7984
SH	−0.141	0.0026	−0.187	0.0040	−0.068	0.3122
R	0.081	0.0842	0.096	0.1428	−0.010	0.8803
UES	−0.050	0.2873	−0.130	0.0470	−0.053	0.4358
UIS	−0.022	0.6341	−0.057	0.3820	−0.082	0.2240
SD	0.095	0.0420	0.002	0.9814	0.116	0.0845
D	0.157	0.0008	0.205	0.0016	0.081	0.2323
V	0.107	0.0227	−0.016	0.8050	0.177	0.0084
SU	−0.018	0.7063	0.034	0.6035	0.048	0.4819
BD	0.132	0.0048	0.182	0.0052	0.063	0.3487
SB	0.273	0.0000	0.373	0.0000	0.153	0.0234

rho: Spearman’s correlation; p: statistical significance. Spearman’s rho correlation coefficient. AC: active coping, P: planning, PR: positive reframing, A: acceptance, SH: sense of humor, R: religion, UES: use of emotional support, UIS: use of instrumental support, SD: self-distraction, D: denial, V: venting, SU: substance use, BD: behavioral disengagement, SB: self-blame.

**Table 5:** The level of perceived stress and strategies of coping with difficult situations (14 scales).

Mini-COPE	The Level of Perceived Stress—PSS-10														*Z*	*p*
	Low/Average							High								
	M	SD	Min.	Max.	Q1	Q2 (Me)	Q3	M	SD	Min	Max.	Q1	Q2 (Me)	Q3		
**General *n* = 456**																
AC	2.25	0.56	0.00	3.00	2.00	2.00	2.50	2.27	0.57	0.00	3.00	2.00	2.00	3.00	−0.51	0.6114
P	2.12	0.54	0.00	3.00	2.00	2.00	2.50	2.14	0.63	0.00	3.00	1.75	2.00	2.50	−0.63	0.5297
PRE	1.87	0.58	0.50	3.00	1.50	2.00	2.00	1.74	0.71	0.00	3.00	1.50	2.00	2.00	−2.14	0.0326
A	1.87	0.59	0.00	3.00	1.50	2.00	2.00	1.86	0.66	0.00	3.00	1.50	2.00	2.00	−0.14	0.8873
SH	0.99	0.57	0.00	2.50	0.50	1.00	1.50	0.84	0.63	0.00	2.50	0.50	1.00	1.50	−2.55	0.0108
R	1.31	0.88	0.00	3.00	0.50	1.00	2.00	1.39	0.94	0.00	3.00	0.50	1.50	2.00	−1.05	0.2950
UES	1.82	0.73	0.00	3.00	1.50	2.00	2.00	1.68	0.78	0.00	3.00	1.00	2.00	2.00	−1.64	0.1008
UIS	1.64	0.74	0.00	3.00	1.00	1.50	2.00	1.57	0.80	0.00	3.00	1.00	1.50	2.00	−0.85	0.3935
SD	1.75	0.68	0.00	3.00	1.50	2.00	2.00	1.88	0.69	0.00	3.50	1.50	2.00	2.50	−2.20	0.0275
D	0.90	0.71	0.00	2.50	0.50	1.00	1.50	1.03	0.74	0.00	3.00	0.50	1.00	1.50	−1.88	0.0606
V	1.38	0.61	0.00	3.00	1.00	1.50	2.00	1.46	0.63	0.00	3.00	1.00	1.50	2.00	−1.52	0.1288
SU	0.46	0.64	0.00	3.00	0.00	0.00	1.00	0.43	0.66	0.00	3.00	0.00	0.00	1.00	−0.80	0.4243
BD	0.72	0.59	0.00	3.00	0.00	0.50	1.00	0.81	0.62	0.00	2.50	0.00	1.00	1.00	−1.62	0.1058
SB	1.05	0.68	0.00	3.00	0.50	1.00	1.50	1.40	0.78	0.00	3.00	1.00	1.50	2.00	−5.06	0.0000
**Women *n* = 235**																
AC	2.29	0.61	0.00	3.00	2.00	2.50	3.00	2.28	0.54	0.50	3.00	2.00	2.50	2.50	−0.35	0.7290
P	2.17	0.49	0.50	3.00	2.00	2.00	2.50	2.20	0.56	1.00	3.00	2.00	2.00	2.50	−0.44	0.6624
PRE	1.91	0.57	0.50	3.00	1.50	2.00	2.00	1.73	0.69	0.00	3.00	1.25	1.50	2.00	−2.29	0.0221
A	1.97	0.54	0.00	3.00	1.50	2.00	2.00	1.83	0.66	0.00	3.00	1.50	2.00	2.00	−1.56	0.1197
SH	0.94	0.55	0.00	2.50	0.50	1.00	1.50	0.77	0.61	0.00	2.50	0.00	1.00	1.00	−2.23	0.0260
R	1.53	0.93	0.00	3.00	1.00	1.50	2.00	1.54	0.89	0.00	3.00	1.00	1.50	2.00	−0.10	0.9171
UES	2.08	0.67	0.00	3.00	1.50	2.00	2.50	1.80	0.73	0.00	3.00	1.50	2.00	2.00	−2.87	0.0041
UIS	1.92	0.70	0.00	3.00	1.50	2.00	2.50	1.70	0.75	0.00	3.00	1.00	2.00	2.00	−2.19	0.0289
SD	1.92	0.64	0.50	3.00	1.50	2.00	2.50	1.97	0.64	0.50	3.00	1.50	2.00	2.50	−0.73	0.4651
D	0.85	0.73	0.00	2.50	0.00	1.00	1.50	1.11	0.75	0.00	3.00	0.50	1.00	1.50	−2.68	0.0073
V	1.57	0.56	0.00	3.00	1.00	1.50	2.00	1.50	0.57	0.00	3.00	1.00	1.50	2.00	−0.71	0.4790
SU	0.24	0.49	0.00	3.00	0.00	0.00	0.50	0.28	0.53	0.00	3.00	0.00	0.00	0.50	−0.68	0.4964
BD	0.68	0.62	0.00	2.50	0.00	0.50	1.00	0.86	0.64	0.00	2.50	0.50	1.00	1.00	−2.18	0.0293
SB	1.00	0.73	0.00	3.00	0.50	1.00	1.50	1.47	0.82	0.00	3.00	1.00	1.50	2.00	−4.42	0.0000
**Men *n* = 221**																
AC	2.22	0.51	0.50	3.00	2.00	2.00	2.50	2.26	0.61	0.00	3.00	2.00	2.00	3.00	−0.77	0.4438
P	2.08	0.57	0.00	3.00	2.00	2.00	2.50	2.06	0.72	0.00	3.00	1.50	2.00	2.50	−0.02	0.9816
PRE	1.83	0.58	0.50	3.00	1.50	2.00	2.00	1.75	0.73	0.00	3.00	1.50	2.00	2.00	−0.69	0.4889
A	1.80	0.62	0.00	3.00	1.50	2.00	2.00	1.89	0.67	0.00	3.00	1.50	2.00	2.50	−1.24	0.2136
SH	1.02	0.58	0.00	2.50	0.50	1.00	1.50	0.95	0.65	0.00	2.50	0.50	1.00	1.50	−0.88	0.3790
R	1.15	0.82	0.00	3.00	0.50	1.00	1.50	1.18	0.98	0.00	3.00	0.00	1.00	2.00	−0.11	0.9129
UES	1.62	0.72	0.00	3.00	1.00	2.00	2.00	1.51	0.83	0.00	3.00	1.00	1.50	2.00	−0.80	0.4226
UIS	1.44	0.70	0.00	3.00	1.00	1.50	2.00	1.38	0.83	0.00	3.00	1.00	1.50	2.00	−0.50	0.6194
SD	1.62	0.68	0.00	3.00	1.00	1.50	2.00	1.76	0.75	0.00	3.50	1.50	1.75	2.50	−1.45	0.1482
D	0.94	0.70	0.00	2.50	0.50	1.00	1.50	0.91	0.71	0.00	3.00	0.50	1.00	1.50	−0.38	0.7046
V	1.24	0.61	0.00	2.50	1.00	1.50	1.50	1.41	0.71	0.00	3.00	1.00	1.50	2.00	−1.90	0.0571
SU	0.62	0.68	0.00	2.00	0.00	0.50	1.00	0.64	0.76	0.00	3.00	0.00	0.50	1.00	−0.19	0.8529
BD	0.75	0.58	0.00	3.00	0.50	1.00	1.00	0.73	0.59	0.00	2.00	0.00	1.00	1.00	−0.12	0.9031
SB	1.08	0.65	0.00	2.50	0.50	1.00	1.50	1.30	0.72	0.00	3.00	1.00	1.50	2.00	−2.46	0.0141

Mann–Whitney test was used. M: arithmetic mean, SD: standard deviation, Q1: quartile I, Q2 (Me): quartile II (median), Q3: quartile III, p: statistical significance, AC: active coping, P: planning, PR: positive reframing, A: acceptance, SH: sense of humor, R: religion, UES: use of emotional support, UIS: use of instrumental support, SD: self-distraction, D: denial, V: venting, SU: substance use, BD: behavioral disengagement, SB: self-blame.

**Table 6:** The level of perceived stress and strategies of coping with difficult situations depending on assisted reproductive technology (ART) procedure using. (14 scales).

Mini-COPE	The Level of Perceived Stress—PSS-10														*Z*	*p*
	Low/Average							High								
	M	SD	Min.	Max.	Q1	Q2 (Me)	Q3	M	SD	Min.	Max.	Q1	Q2 (Me)	Q3		
**Patients after ART procedures *n* = 183**																
AC	2.24	0.58	0.00	3.00	2.00	2.00	3.00	2.24	0.56	0.00	3.00	2.00	2.00	2.50	−0.14	0.8898
P	2.06	0.49	0.50	3.00	2.00	2.00	2.50	2.14	0.70	0.00	3.00	2.00	2.00	2.50	−1.47	0.1419
PRE	1.84	0.57	0.50	3.00	1.50	2.00	2.00	1.77	0.77	0.00	3.00	1.50	2.00	2.00	−0.62	0.5351
A	1.85	0.60	0.00	3.00	1.50	2.00	2.00	1.86	0.76	0.00	3.00	1.50	2.00	2.50	−0.66	0.5075
SH	1.06	0.60	0.00	2.50	0.50	1.00	1.50	0.81	0.61	0.00	2.50	0.50	0.50	1.50	−2.76	0.0057
R	1.24	0.85	0.00	3.00	0.50	1.00	2.00	1.25	0.92	0.00	3.00	0.50	1.00	2.00	−0.01	0.9909
UES	1.75	0.72	0.00	3.00	1.50	2.00	2.00	1.59	0.84	0.00	3.00	1.00	1.50	2.00	−1.18	0.2392
UIS	1.63	0.71	0.00	3.00	1.00	1.50	2.00	1.41	0.88	0.00	3.00	1.00	1.50	2.00	−1.66	0.0964
SD	1.70	0.71	0.00	3.00	1.00	1.75	2.00	1.84	0.71	0.00	3.00	1.50	2.00	2.50	−1.64	0.1000
D	1.07	0.70	0.00	2.50	0.50	1.00	1.50	1.08	0.78	0.00	3.00	0.50	1.00	1.50	−0.11	0.9091
V	1.42	0.58	0.00	3.00	1.00	1.50	2.00	1.41	0.64	0.00	3.00	1.00	1.50	2.00	−0.18	0.8609
SU	0.54	0.67	0.00	3.00	0.00	0.00	1.00	0.31	0.56	0.00	2.50	0.00	0.00	0.50	−2.72	0.0065
BD	0.78	0.62	0.00	2.50	0.00	1.00	1.00	0.71	0.61	0.00	2.50	0.00	1.00	1.00	−0.77	0.4422
SB	1.05	0.70	0.00	3.00	0.50	1.00	1.50	1.33	0.76	0.00	3.00	1.00	1.50	2.00	−3.04	0.0023
**Patients without ART procedures *n* = 273**																
AC	2.25	0.55	0.50	3.00	2.00	2.00	2.50	2.30	0.57	0.50	3.00	2.00	2.50	3.00	−0.56	0.5764
P	2.16	0.57	0.00	3.00	2.00	2.00	2.50	2.15	0.59	0.50	3.00	1.50	2.00	2.50	−0.44	0.6588
PRE	1.88	0.59	0.50	3.00	1.50	2.00	2.00	1.72	0.67	0.00	3.00	1.50	2.00	2.00	−2.22	0.0266
A	1.89	0.59	0.00	3.00	1.50	2.00	2.00	1.85	0.60	0.00	3.00	1.50	2.00	2.00	−0.85	0.3934
SH	0.93	0.54	0.00	2.50	0.50	1.00	1.00	0.86	0.64	0.00	2.50	0.50	1.00	1.50	−0.95	0.3420
R	1.36	0.91	0.00	3.00	0.75	1.50	2.00	1.48	0.95	0.00	3.00	1.00	1.50	2.00	−1.18	0.2375
UES	1.87	0.74	0.00	3.00	1.50	2.00	2.50	1.73	0.74	0.00	3.00	1.00	2.00	2.00	−1.38	0.1691
UIS	1.65	0.76	0.00	3.00	1.00	1.50	2.00	1.66	0.73	0.00	3.00	1.00	1.50	2.00	−0.12	0.9033
SD	1.78	0.65	0.00	3.00	1.50	2.00	2.00	1.91	0.69	0.00	3.50	1.50	2.00	2.50	−1.48	0.1397
D	0.78	0.70	0.00	2.50	0.00	0.50	1.00	1.00	0.72	0.00	3.00	0.50	1.00	1.50	−2.66	0.0078
V	1.36	0.64	0.00	3.00	1.00	1.50	2.00	1.50	0.62	0.00	3.00	1.00	1.50	2.00	−1.80	0.0713
SU	0.40	0.61	0.00	2.00	0.00	0.00	0.75	0.50	0.70	0.00	3.00	0.00	0.00	1.00	−1.14	0.2559
BD	0.68	0.57	0.00	3.00	0.00	0.50	1.00	0.87	0.63	0.00	2.50	0.50	1.00	1.00	−2.73	0.0063
SB	1.05	0.68	0.00	2.50	0.50	1.00	1.50	1.45	0.80	0.00	3.00	1.00	1.50	2.00	−4.03	0.0001

Mann–Whitney test was used. M: arithmetic mean, SD: standard deviation, Q1: quartile I, Q2 (Me): quartile II (median), Q3: quartile III, p: statistical significance, AC: active coping, P: planning, PR: positive reframing, A: acceptance, SH: sense of humor, R: religion, UES: use of emotional support, UIS: use of instrumental support, SD: self-distraction, D: denial, V: venting, SU: substance use, BD: behavioral disengagement, SB: self-blame.

## Data Availability

The data analyzed in the study are available upon request to the corresponding author.
